# Thermally Tunable Dynamic and Static Elastic Properties of Hydrogel Due to Volumetric Phase Transition

**DOI:** 10.3390/polym12071462

**Published:** 2020-06-30

**Authors:** Yuqi Jin, Teng Yang, Shuai Ju, Haifeng Zhang, Tae-Youl Choi, Arup Neogi

**Affiliations:** 1 Department of Physics, University of North Texas, P.O. Box 311427, Denton, TX 76203, USA; yuqijin@my.unt.edu; 2 Department of Mechanical and Energy Engineering, University of North Texas, 3940 North Elm Suite F101, Denton, TX 76207, USA; tengyang@my.unt.edu (T.Y.); shuaiju@my.unt.edu (S.J.); haifeng.zhang@unt.edu (H.Z.); tae-youl.choi@unt.edu (T.-Y.C.)

**Keywords:** mechanical properties, bulk modulus, density, deswelling ratio, Poisson’s ratio thermo-responsive, gels, polymer hydrogels, temperature sensitive, dynamic elasticity

## Abstract

The temperature dependence of the mechanical properties of polyvinyl alcohol-based poly n-isopropyl acrylamide (PVA-PNIPAm) hydrogel was studied from the static and dynamic bulk modulus of the material. The effect of the temperature-induced volumetric phase transition on Young’s Modulus, Poisson’s ratio, and the density of PVA-PNIPAm was experimentally measured and compared with a non-thermo-responsive Alginate hydrogel as a reference. An increase in the temperature from 27.5 to 32 °C results in the conventional temperature-dependent de-swelling of the PVA-PNIPAm hydrogel volume of up to 70% at the lower critical solution temperature (LCST). However, with the increase in temperature, the PVA-PNIPAm hydrogel showed a drastic increase in Young’s Modulus and density of PVA-PNIPAm and a corresponding decrease in the Poisson’s ratio and the static bulk modulus around the LCST temperature. The dynamic bulk modulus of the PVA-PNIPAm hydrogel is highly frequency-dependent before the LCST and highly temperature-sensitive after the LCST. The dynamic elastic properties of the thermo-responsive PVA-PNIPAm hydrogel were compared and observed to be significantly different from the thermally insensitive Alginate hydrogel.

## 1. Introduction

Hydrogels were first discovered in 1894 [1] as a cross-linked polymer that swells in water [2]. The pH and temperature-sensitive phase transition effect of poly N-iso-propylacrylamide hydrogel were observed due to the hydrophobic bond formation [3]. The activities related to the hydrogels were mainly focused on the water absorbing properties of simple cross-linked synthetic polymer networks [4]. Around the 1970s, polyethylene oxide (PEO) drew more attention due to its abundance and higher variations in terms of molecular weight [5]. This led to the study of volumetric phase transition in hydrogel [6] by temperature [7], light [8], ionic strength [9], and pH [10]. The phase change in poly(N-isopropylacrylamide) (pNIPAAm) is observed around 32 °C and is a potentially useful material for biomedical application [11]. Bulk Poly (Vinyl Alcohol) (PVA) Poly (N-isopropyl acrylamide) (PNIPAm) composites are one of the thermally responsive hydrogels [12]. PVA-PNIPAm hydrogel has a clear phase transition between 30 and 35 °C, which is called lower critical solution temperature (LCST). Below LCST the cross-linked polymer is filled with water. However, the water molecules are expelled from the PNIPAm network after LCST. The phase transition of the tunable hydrogel is usually reversible, which introduced broad categories of application.

The mechanical properties of hydrogels have been gaining increasing importance due to their uses in areas such as soft-robotics [13,14], sensing [15], and biomedical applications [16,17,18]. The 2-hydroxyethyl methacrylate (HEMA)–acrylic acid (AA) comonomer is found to have a different Young’s Modulus from PNIPAM with a pH-dependent mechanical property [12]. The volumetric control and variation of mechanical properties of the hydrogel is also relevant for unique applications such as agricultural deep percolation [19], temperature-sensitive micropumps [20], and tunable phononics [21,22]. Many patents of stimulus sensitive hydrogels were proposed for applications in wound dressing [23] and drug delivery [24]. The mechanical properties of PVA hydrogel could be tuned at different temperature and can be used in mini-invasive surgery [25]. Mechanical properties of the PNIPAm hydrogel are characterized before and after phase transition in terms of either elasticity or plasticity. From tensile tests, a 5% PNIPAm hydrogel exhibits a Young’s Modulus of about 45 kPa and strength about 420 kPa before the phase transition [26]. A PNIPAm hydrogel system exhibits a change in its Young’s Modulus from 35 to 65 kPa and an accompanying tensile strength change from 350 to 650 kPa due to a change in the ratio of PNIPAm and cross-linker [27]. The tensile tested modulus difference of the hydrogel before the lower critical solution temperature (LCST) and after the LCST could reach 20 times in a non-reinforced PNIAPm hydrogel [28]. A nano-cellulose reinforced PNIPAm has shown to exhibit a 20-fold difference in its modulus before and after the LCST and about seven fold difference without any reinforcement [29]. 

The existing characterization of the mechanical properties of the PNIPAm hydrogel are mostly focused on thermo-sensitive polymers especially on the variation of the percentage of cross-linker without a standard hydrogel as reference. The tensile tests were mostly not environmentally controlled as the hydrogels usually have a tendency to dehydrate during the tests. In this report, the temperature-dependent static mechanical properties of PVA-PNIPAm hydrogel are presented using a compression testing method. A regular Alginate hydrogel (Hydrogel^®^ N) was used as a reference as it does not undergo a phase transition due to temperature variation in an environmentally controlled chamber under identical compressing tests. 

Ultrasonic waves can be used for non-destructive evaluation of elasticities in many materials such as metals because the microstructure inside the metals or alloys is much smaller than the ultrasound wavelength at the operating frequency. The samples can be considered to be a homogeneous media for the ultrasonic waves and its dynamic modulus estimated from the ultrasound evaluations has almost similar values as its static modulus estimated from the mechanical tests. However, in hydrogels and especially in hydrogel composites, the elasticity is highly dependent on the frequency of evaluation due to the smaller difference in the size of the microstructure compared to the probing wavelength. The estimated values of the static elastic constant can be significantly lower than the high frequency dynamic elasticity and can vary over several orders of magnitudes [30]. Current work is limited to the evaluation of the Young’s Modulus that is estimated from the speed of longitudinal and shear modes of ultrasonic waves in the medium. This evaluation requires that the longitudinal wave transducer and shear wave transducer be at the same fundamental frequency with identical frequency bandwidth. However, it is challenging to find longitudinal and shear wave transducers that operate at the same central frequency and the same bandwidth. Since the dynamic elasticity is highly frequency-dependent in hydrogels, shear wave and longitudinal wave sound velocities at different frequencies cannot be used to estimate the Young’s Modulus. Another limitation, especially in PVA-PNIPAm composites hydrogel, is the high volume of aqueous water molecules. The liquid water medium does not support shear mode for accurate measurement of the shear modulus of a water-based composite. Using the shear wave speed of sound in 5% PVA-PNIPAm hydrogel with over 90% water molecule thereby introduces additional errors in dynamic modulus measurements. To overcome these limitations, only longitudinal waves were used to measure the frequency-dependent speed of sound for the estimation of dynamic bulk modulus as it is dependent on the longitudinal mode of vibrations. 

In this study, the novel temperature and frequency-dependent dynamic elasticity were first experimentally obtained from static and dynamic measurement on thermal tunable PVA-PNIPAm hydrogel. Comparing with a non-thermal tunable and less frequency-dependent Alginate hydrogel, the dynamic elasticity of PVA-PNIPAm showed up to 174% more tunability by varying temperature and operating frequency than the reference Alginate hydrogel. The soft PVA-PNIPAm hydrogel could be barely used for mechanical applications due to its low stiffness. However, in this study, the investigated temperature and frequency-dependent dynamic elasticity show its potential for acoustic or ultrasonic applications. 

## 2. Experimental Setup and Methods

The first part of the experiments demonstrates the temperature-dependent low strain-rate mechanical tests compared to the static mechanical properties as well as the density changes in the thermally tunable PVA-PNIPAm hydrogel and thermally insensitive Alginate hydrogels.

The second part of the experiments presents the continuous compression tests (in contact mode) of a PVA-PNIPAm hydrogel disc sample during a reversible thermal cycle around the phase transition temperature. This experiment demonstrates that the PVA-PNIPAm hydrogel in this study was thermally reversible.

The third part of the experiment is the high frequency dynamic elasticity test using non-contact or ultrasonic inspection. Due to the strong frequency-dependent sound wave dispersion in the hybrid composite PVA-PNIPAm, different dynamic incompressibility was observed at various operating frequencies. This frequency-dependent temperature-sensitive behavior was also compared to a reference Alginate hydrogel as this material system does not undergo volumetric phase transition.

### 2.1. Temperature-Dependent Static Elasticity Measurement

**Figure 1:**
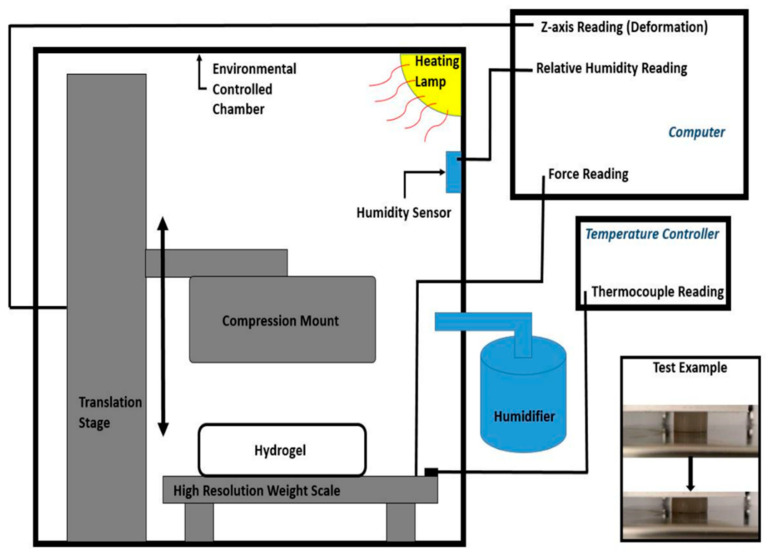
Experimental setup of environmentally controlled compression test. The environmentally controlled chamber offered at least 96% relative humidity in the chamber with a humidifier to minimize the unexpected dehydration of the testing polymer sample. The heating lamp was controlled by a temperature controller which collaborated with a calibrated thermocouple monitoring the temperature inside of the chamber. The bottom figure within the inset shows the disc-shaped PVA-PNIPAm sample before and after compression.

As the Figure 1 shows, an environmentally controlled closed chamber was used for the temperature-dependent Young’s Modulus and Poisson ratio measurement. Highly sensitive small force load cells were utilized to record the compression force on the hydrogel disc samples. Alternatively, Mettler Toledo XS205 Dual Range weight scale was used as a high-sensitive, high-resolution load cell (10 μm resolution and a maximum capacity 2 N). A Thorlabs MTS50/M-Z8 linear translation stage with 10 μm resolution was attached with a 3D-printed compression mount to provide the compression force to the disc-cylinder shape hydrogel specimens. The force and linear displacement were recorded during the operations within the experiment. Young’s Modulus was estimated from the linear stress–strain ratio. A high-resolution camera was used to measure the height deformation and the extension of the specimens for the Poisson ratio measurement. The bulk modulus was estimated from the Young’s Modulus and Poisson ratio. A heating lamp was used to heat up the air in the environmentally controlled chamber to a desired temperature. To avoid water evaporation of the specimens due to heating, relative humidity in the chamber was maintained as high as possible. The humidity was maintained at 95% or above the relative humidity of the environmentally controlled chamber with the temperature maintained below 36 °C. The ambient air temperature and relative humidity condition were sensed using an Arduino programmed DHT-22 sensor within the closed chamber. A thermocouple was placed between the specimens connected to a temperature controller to monitor the plate on the weight scale. Compression tests were performed at least five times for different sizes of specimens at 22.4, 28, 31, 34 and 39 °C (±0.2 °C).

### 2.2. Mechanical Test Method Verification

The reliability of the compression test was standardized using a soft solid-state polymer (Ecoflex 00-05 Platinum cure Silicone rubber) with known Young’s Modulus (82 KPa). Before the self-assembled high accurate compression test machine being used for examining elasticity, the known elasticity Silicone rubber was made to verify the setup. The silicone rubber was made by Ecoflex 00-05 component A and B. The two viscos fluids were mixed well with a volume ratio 1:1. After mixing, the mixture was placed in a vacuum to minimize the elasticity impact by air bubbles. The results of the Young’s Modulus from the silicone rubber were found to highly agree with the known value from the product data sheet with 1.2% error.

### 2.3. Temperature-Dependent Density and Volumetric Deswelling Ratio Measurements

The temperature-dependent volume of the specimens was estimated from the diameter and height determined by ImageJ after calibration using a standardized target. A high-resolution camera was set up in front of the specimens to record the change in height and diameter of the specimens with a change in temperature. The volumetric deswelling ratio was determined from the ratio between the changes in volume of the specimen at a certain temperature from its original volume. The same method was used to find the volume of specimens for the temperature-dependent density measurement. Each specimen of hydrogel was heated up slowly from 22.4 °C (room temperature) to 39 °C by about 0.7 °C/min. At each temperature point, four specimens were sampled. The temperature of the specimens was monitored with a calibrated thermocouple and a temperature controller. The mass was measured by Mettler Toledo XS205 Dual Range high-resolution weight scale at 22.4, 28, 31, 34 and 39 °C (±0.2 °C). The density was determined from the mass–volume ratio. 

### 2.4. Temperature and Frequency-Dependent Dynamic Bulk Modulus Test

**Figure 2:**
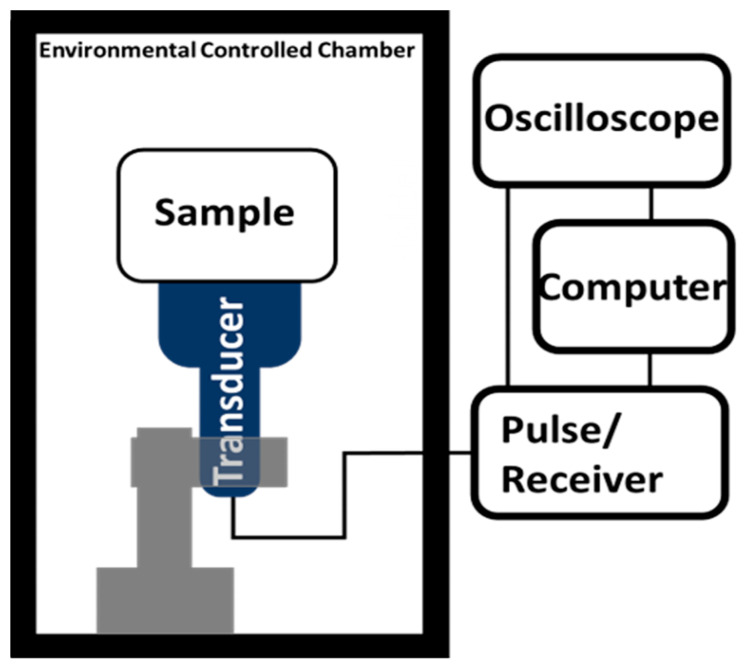
Experimental setup of environmentally controlled ultrasound velocity test. The chamber temperature and humidity were still controlled by heating lamp and humidifier. The ultrasound transducer was pre-calibrated working under various temperatures and produced consistent results in the temperature range in this study.

The temperature-dependent dynamic bulk modulus was obtained from temperature density measurement and temperature and frequency-dependent speed of sound measurement as expressed as $K_{\left(T,f\right)}=\rho_{\left(T\right)}C_{\left(T,f\right)}$, where $K_{\left(T,f\right)}$ is the dynamic bulk modulus results dependent on hydrogel temperature and operating frequency, $\rho_{\left(T\right)}$ is the temperature-dependent density acquired by the method listed in Section 2.3. $C_{\left(T,f\right)}$ is the temperature and frequency-dependent speed of sound measurement from environmentally controlled ultrasound time of flight measurement [22] as Figure 2 shows. The ultrasound velocity was calculated from $C_{\left(T,f\right)}=2d/t_{\left(T,f\right)}$, where d is the thickness of hydrogel disc samples. 

### 2.5. Materials

The PVA-PNIPAm (polyvinyl alcohol-based poly n-isopropyl acrylamide) hydrogel composites were produced using a monomer solution with a weight ratio of 0.1 of NIPA (poly n-isopropyl acrylamide) monomer (TCI Chemicals, Tokyo, Japan), 0.02 of N’-methyene-biacrylamide crosslinker (BIS; 2-methacryloxyethyl, Polysciences Inc., Warrington, PA, USA), and 0.84 of DI water. Poly (Vinyl Alcohol) (PVA) (Polysciences Inc.) and magnetic nanoparticles were added to the monomer solution by a weight ratio of 0.01 and 0.02 of the total weight. The magnetic nanoparticles were stirred for more than 24 h to attain a homogeneous solution. The solution was then heated to 50 °C for dissolving PVA in the solution. The solution of the mixture was then cooled down using an ice bath in the presence of N2 (Dinitrogen) gas for more than our hour to remove the absorbed oxygen from the solution. To initiate and accelerate crystallization of the bulk gel (polymer), ammonium persulfate and TEMED (Tetramethylethylenediamine) was used. The gel that was finally produced was placed in the DI water for more than two days to keep it hydrated. Because PVA is soluble in water, it is necessary to replace the PVA water solution by DI water in the bulk hydrogel. Alginate hydrogels were made from Hydrogel^®^ N Alginate hydrogel powder dissolved in water at 45 °C by weight percentage ratios of 6%, 10% and 14%. The mixture was stirred for 25 min. The solutions were cooled down to room temperature to obtain the Alginate hydrogels.

## 3. Results and Discussion

**Figure 3:**
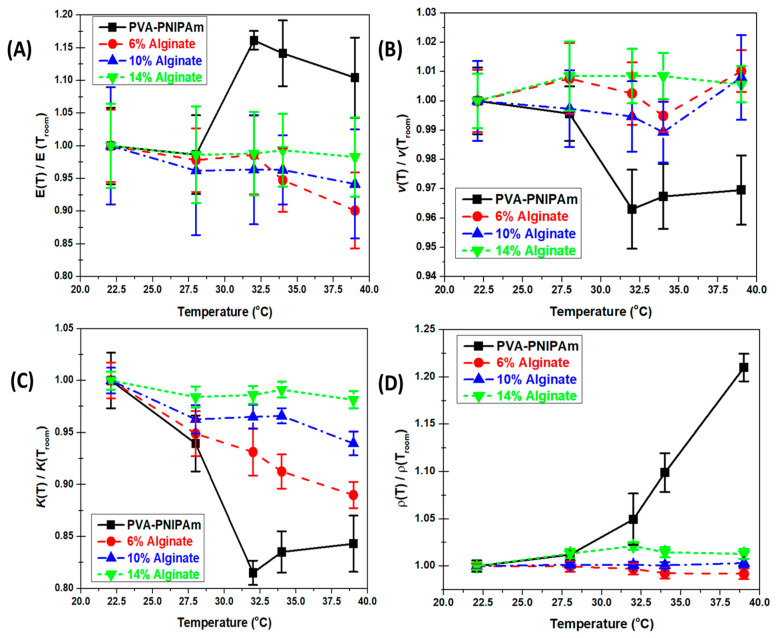
Temperature-dependent compression test results of PVA-PNIPAm hydrogel and Alginate hydrogel preformed in the environmentally controlled chamber. (**A**) Normalized temperature-dependent Young’s Modulus behavior comparison between PVA-PNIPAm hydrogel and Alginate hydrogel. (**B**) Normalized temperature-dependent Poisson’s ratio behavior comparison between PVA-PNIPAm hydrogel and Alginate hydrogel. (**C**) Normalized temperature-dependent bulk modulus behavior comparison between PVA-PNIPAm hydrogel and Alginate hydrogel. (**D**) Normalized temperature-dependent density behavior comparison between PVA-PNIPAm hydrogel and Alginate hydrogel. The normalization of each data line was based on the room temperature properties.

The temperature-dependent mechanical property results of PVA-PNIPAm and Alginate hydrogel were normalized using the measured values at room temperature in order to compare the temperature-dependent mechanical properties’ varying behavior. Figure 3A shows the temperature-dependent Young’s Modulus (Figure 3A) of the hydrogel materials. The normalization factors of the curves were 14.780 kPa for PVA-PNIPAm, 25.694 kPa for 6% Alginate hydrogel, 65.146 kPa for 10% Alginate hydrogel, and 151.759 kPa for 14% Alginate hydrogel. In the temperature-dependent Poisson’s ratio plot (Figure 3B), the normalization factors of the curves were 0.46 for PVA-PNIPAm, 0.394 for 6% Alginate hydrogel, 0.375 for 10% Alginate hydrogel, and 0.355 for 14% Alginate hydrogel. In the temperature-dependent Bulk Modulus plot (Figure 3C), the normalization factors of the curves were 61,583 Pa for PVA-PNIPAm, 9857 Pa for 6% Alginate hydrogel, 23,689 Pa for 10% Alginate hydrogel, and 55,999 Pa for 14% Alginate hydrogel. In the temperature-dependent density plot (Figure 3D), the normalization factor of the curves were $1048.9\;\mathrm{kg}/m^{3}$ for PVA-PNIPAm, $1257.3\;\mathrm{kg}/m^{3}$ for 6% Alginate hydrogel, $1409.2\;\mathrm{kg}/m^{3}$ for 10% Alginate hydrogel, and $1410.8\;\mathrm{kg}/m^{3}$ for 14% Alginate hydrogel. The summarized normalization factors were listed in Table A1.

In our previous study [22], the LCST of the PVA-PNIPAm hydrogel also acted as the transition point for sound velocity and dispersion. The sound velocity had a sharp increase when the temperature of the PVA-PNIPAm hydrogel was increased beyond the LCST. The dispersion effect of sound showed a more significant change during the phase transition. Based on the dispersion of sound, the potential for conducting and applying the frequency-dependent dynamic elasticity motivated the current stage of the study on the temperature and frequency-dependent dynamic elasticity.

The temperature-dependent Young’s Modulus measured from the compression test is shown in Figure 3A. The stiffness of the PVA-PNIPAm hydrogel composites generally shows an increase with the temperature rise. However, as Figure 3B shows, the temperature-dependent Poisson ratio behavior is nearly opposite to the trend in Young’s Modulus. The Poisson ratio change is more similar to the temperature-induced change in the density of the gel due to volumetric phase transition. The trend is also similar to the characteristics change of the temperature-dependent bulk modulus. The lower critical solution temperature (LCST) point of PVA-PNIPAm hydrogel is between 31 and 34 °C. The Young’s Modulus attains a maximum value at 34 °C and undergoes an increase of 15% between 22.4 and 39 °C. At room temperature, a part of the polymer chains in PVA-PNIPAm hydrogel is hydrophilic in nature and changes to hydrophobic at the LCST temperature. The specimens have a liquid–liquid phase below the LCST temperature and exhibit a very high Poisson ratio with strain approaching 35%. As temperature is raised, the crosslink between polymer chains and bonded water molecules start to break around 28 °C. The polymer chains bond with each other and the Young’s Modulus significantly increases from 28 to 31 °C as the bulk modulus reduces correspondingly at this temperature range. Since Alginate hydrogel is not sensitive to temperature, the mechanical properties were determined using different concentrations of the Alginate concentration compared to the PVA-PNIPAm material system. Figure 3A shows that the Young’s Modulus for the Alginate hydrogel is unaffected by any change in temperature although the magnitude increases its concentration. 

Above the LCST, the PVA-PNIPAm hydrogel undergoes a volumetric phase transition and decreases in overall size and averaged domain size [31,32,33]. The Poisson ratio of the hydrogel is reduced rapidly from 28 to 34 °C and the stiffness of the specimens changes from jelly-like to a more rubber-like form and shape. As in case of Young’s Modulus, the Poisson ratio of the Alginate hydrogel is not significantly influenced by the change in temperature. In the temperature-dependent Young’s Modulus and bulk modulus results, the Alginate hydrogel has a limited drop due to thermal softening as it is in not a thermally sensitive hydrogel. Compared to the sharp change in Young’s Modulus of PVA-PNIPAm, the Young’s Modulus of Alginate hydrogel was observed to be unaffected due to a change in temperature. The larger elasticity drop was observed in lower concentration Alginate hydrogel. In a temperature-dependent Poisson’s ratio, the Alginate hydrogel showed a stable property which was not affected by temperature in the range from room temperature to 39 °C. 

Bulk PVA-PNIPAm hydrogel undergoes a volumetric phase transition at about 32.5 °C due to the cooperative change of the cross-linker in the polymer molecules. Before LCST, the polymer chains are partially cross-linked with free water. The composite shows a lower mechanical properties. The hydrogel polymer network changes from hydrophilic to hydrophobic nature expelling the water from the PNIPAm polymer network. At the LCST, the phase separation results in the PNIPAm chains and their conformation changes from coiled to globular due to the increase in temperature. The intra- and inter-molecular hydrogen bonds form between the amide groups during the coil-to-globule transition. The coil-to-globule transition leads to the change in the elastic properties of the polymer. After the transition to globular chains, the composite shows an increase in mechanical strength.

**Figure 4:**
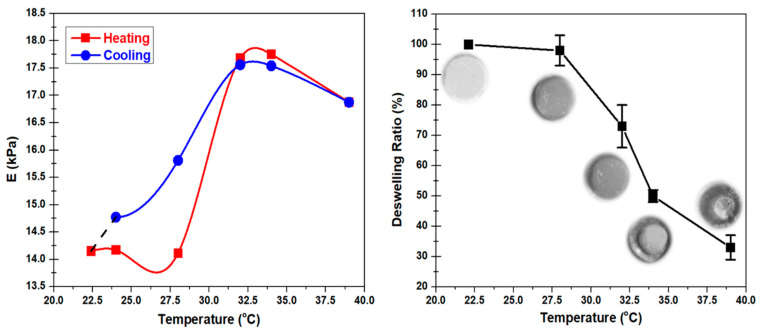
Temperature-dependent Continuous compression test and density measurement results of PVA-PNIPAm hydrogel preformed in the environmental controlled chamber. (**Left**) Continuous compressing test of one PVA-PNIPAm hydrogel disc sample under heating up and cooling down processes. (**Right**) Deswelling ratio of PVA-PNIPAm hydrogel calculated from measured weight and volume of PVA-PNIPAm samples. The subfigures are the photographs of one PVA-PNIPAm hydrogel disc under different temperature points.

The density of the hydrogel also increases with the temperature within the environmental chamber (Figure 3D). The density of the hydrogels, including the PVA-PNIPAm hydrogel, does not change much below 28 °C. As the PVA-PNIPAm hydrogel changes from a hydrophilic state to the hydrophobic state between 28 to 31 °C, the density increases as the water is expelled out of the polymer network. The color of hydrogel specimens changed from light-milky to white (Figure 4, right). Beyond 34 °C, the dehydration rate of hydrogel specimens decreases with a clearly visible sign of volumetric deswelling which leads to large density increase. The color of the specimens changed to semi-transparent which is close to silicon rubber. The density of Alginate hydrogel increases rapidly from 6% to 10% Alginate concentration, but increase a little from 10% to 14%. The density of the Alginate is therefore not influenced by its temperature within the range from room temperature to 39 °C. Overall, for conventional Alginate hydrogel, the mechanical properties can only be altered by concentration, which limits its potential applications. 

The temperature-dependent low strain rate mechanical tests of thermal tunable PVA-PNIPAm hydrogel and non-tunable Alginate hydrogel, showed expected modification in the static elasticity of the PVA-PNIPAm hydrogel around 20% by increasing the temperature about 17 °C. This behavior in elasticity change was not observed in Alginate hydrogel. In the temperature-dependent Young’s Modulus and bulk modulus results, the Alginate hydrogel has a limited drop due to thermal softening. It is not a temperature-tunable hydrogel. Comparing a sharp change in Young’s Modulus of PVA-PNIPAm, the Young’s Modulus of Alginate hydrogel was considered to have no change.

Figure 4 shows the effect of thermal cycling on the hydrogel. Figure 4 (left) shows the result of the continuous compression test within the small strain (3%) limit subjected to a low strain rate (0.05 mm/min) while heating and cooling the sample. Beyond LCST, during the cooling process, the Young’s Modulus at 31 and 34 °C were lower than the Young’s Modulus values during heating at the same temperature points. Below the LCST, the determined behavior was the opposite. The temperature-dependent Young’s Modulus values at 22.4 and 28 °C during the cooling process were significantly higher than the values during the heating process at the same temperature points. The interesting behavior found in this experiment showed the possibility of soft robotic application on PVA-PNIPAm hydrogel that can be controlled by the thermal cycling process. The deformation of the hydrogel with a constant stress undergoes a clear decrease during natural cooling from the LCST after the hydrogel has heated beyond its LCST. The thermally powered change in deformation can lead to a finite amount of mechanical work done in this medium. 

For PVA-PNIPAm hydrogel, the concentration is temperature-dependent, as well as its mechanical properties. However, for Alginate hydrogel, the mechanical properties are significantly changed by altering the concentration. From 6% to 14% Alginate concentration, the Young’s Modulus increases approximately seven times. Therefore, even though Alginate hydrogel is not temperature tunable, it can be used in some applications requiring stable environmental temperature, such as plant-root water absorption [19] and contact lens [34]. In these applications, temperature-stable properties are preferred.

Comparing with Alginate hydrogel, the mechanical properties of PVA-PNIPAm hydrogel are significantly different as the environmental temperature changes. When the temperature increases from 28 to 34 °C, the Young’s Modulus of PVA-PNIPAm hydrogel increases by 15%, implying a stiffer hydrogel. At 34 °C, the Poisson’s ratio decreases by 30% but its density increases by 3% compared to that at room temperature. The volumetric deswelling ratio of PVA-PNIPAm hydrogel increases from 0% to 70% as the temperature increases from room temperature (22.4 °C) to 39 °C, implying a decrease in volume by 70%. The net volumetric swelling change for a pH-sensitive antigen–antibody semi-IPN hydrogel [35] is about 10% of its original volume. The net volumetric swelling capabilities of temperature-sensitive PVA-PNIPAm hydrogel is 70% of its original volume, which is 7 times bigger than antigen–antibody semi-IPN hydrogel. Moreover, from 22.4 to 39 °C, the net volumetric swelling change for N-isopropylacrylamide/chlorophyllin copolymer gel [36], sensitive to temperature and light, is approximate 34% of original volume, which is two times smaller than PVA-PNIPAm hydrogel. This higher deswelling phenomenon makes PVA-PNIPAm hydrogel especially useful in biomedical applications, such as tissue engineering [14], mini-invasive surgery [25], 4D bio-printing [37], swelling-controlled drug delivery [38], and biomedical soft robots [39]. Other potential applications for PVA-PNIPAm hydrogel includes Microfluidic actuator [40], self-folding devices [41], and tunable micro-lenses [42]. In the future study, the dynamic modulus such as effective bulk modulus and effective density could be directly measured from some of the recently invented techniques [43,44] to relate the static modulus from invasive machine tests to dynamic modulus from non-invasive acoustic measurements. 

The continuous compression tests in contact mode of a PVA-PNIPAm hydrogel disc sample during a reversible thermal cycle around the phase transition temperature showed that the PVA-PNIPAm hydrogel in this study was thermally reversible Due to the strong frequency-dependent sound wave dispersion in the hybrid composite PVA-PNIPAm, different dynamic incompressibility was observed at various operating frequency. By introducing the thermal induced phase change of the PVA-PNIPAm into the frequency-dependent elastic properties, the tunability of the dynamic elasticity (174%) was shown to be more than 8 times than its static elasticity. This frequency-dependent temperature sensitive behavior was also compared to a reference Alginate hydrogel as this material system does not undergo volumetric phase transition.

The temperature and frequency-dependent dynamic bulk modulus was obtained from the temperature-dependent density measurement and temperature and frequency-dependent speed of longitudinal sound measurement by equation $K_{\left(T,f\right)}=\rho_{\left(T\right)}C_{\left(T,f\right)}$. The longitudinal sound velocity at various frequencies around lower ultrasound range was measured as different temperatures. Additionally, the speed of sound also increased when the temperature of the PVA-PNIPAm rose. In order to have a contour map of the fully temperature and frequency-dependent elasticity behavior, the longitudinal sound velocity was measured from 0.1 MHz to 1 MHz at different temperature points.

**Figure 5:**
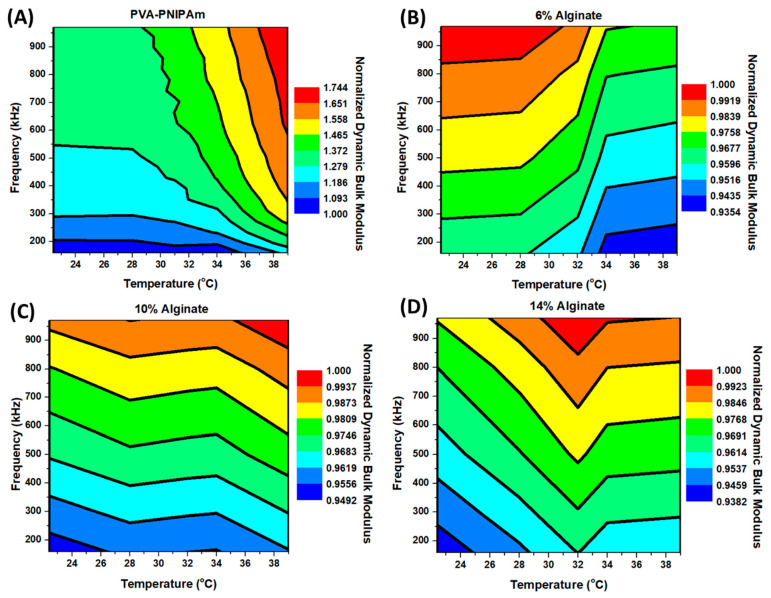
Temperature and frequency-dependent dynamic bulk modulus calculated by temperature and frequency-dependent longitudinal sound velocity and temperature-dependent density. (**A**) Temperature and frequency-dependent dynamic bulk modulus of PVA-PNIPAm hydrogel. (**B**) Temperature and frequency-dependent dynamic bulk modulus of 6% Alginate hydrogel. (**C**) Temperature and frequency-dependent dynamic bulk modulus of 10% Alginate hydrogel. (**D**) Temperature and frequency-dependent dynamic bulk modulus of 14% Alginate hydrogel. The values of dynamic bulk modulus were normalized by the dynamic bulk modulus values of each hydrogel at 100 kHz and 22.4 °C. The normalized factors were 1.650 GPa in (**A**), 2.024 GPa in (**B**), 2.877 GPa in (**C**) and 3.005 GPa in (**D**).

Figure 5 shows the temperature and frequency-dependent dynamic bulk modulus calculated from speed of sound measurements. A comparison between PVA-PNIAPm and Alginate hydrogel was performed within the same temperature and frequency ranges. In Alginate hydrogels, temperature and frequency-dependent dynamic bulk modulus difference in the experimented range were smaller than 8%. The dynamic modulus of 10% and 14% Alginate hydrogel is clearly frequency-dependent but not highly temperature-dependent. From the results of 4% Alginate hydrogel, the dynamic modulus still increases with an increase in the actuating frequency, but due to thermal softening effect, the dynamic bulk modulus shows a slightly slow dynamic incompressibility at low frequency and high temperature, and higher dynamic bulk modulus at high frequency and room temperature. However, unlike Alginate hydrogels, the dynamic bulk modulus in PVA-PNIPAm hydrogel is almost doubled at high frequencies and higher temperature compared to that at low frequency and below the LCST. Before the LCST, the dynamic bulk modulus values of PVA-PNIPAm are more frequency-dependent and less temperature-dependent. Once the temperature increases beyond LCST, the dynamic bulk modulus values are more temperature sensitive and less dependent on the operating frequency. The interesting behavior occurs from the change in microstructure size within the PVA-PNIPAm hydrogel network before and after the LCST. PVA-PNIPAm is closer to a solid–liquid composite material at the operating frequency range, and it becomes more like a homogeneous solid material beyond LCST. Thus, the mechanical properties of the PVA-PNIPAm will depend not only on its temperature, but also on the frequency of the operation. Most of the prior studies have been performed without considering the dispersion of the mechanical vibrations or the ultrasonic waves through the medium. The contour map of the dynamic bulk modulus of PVA-PNIPAm provides a novel characterization tool to investigate thermal-tunable composites hydrogel-dependent on both temperature and frequency. 

From the high-frequency dynamic elasticity test, the outstanding tunability of the PVA-PNIPAm was clearly demonstrated. Due to the strong dispersion or frequency-dependent sound velocity in the hybrid composite PVA-PNIPAm, the thermally sensitive polymer behaved in different dynamic incompressibility along with various operating frequencies. By introducing the thermal tunability of PVA-PNIPAm into the frequency-dependent elasticity, the tunability of the dynamic elasticity (174%) was observed to be more than eight times its static elasticity. This dynamic elasticity was less than 8% in a reference Alginate hydrogel.

## 4. Conclusions

In this study, both static and dynamic elastic properties of PVA-PNIPAm hydrogel and Alginate hydrogel are investigated. The static and dynamic elasticity of the reference Alginate hydrogel does not change much with temperature and can be used for non-stimulus applications like water-absorption. On the other hand, the mechanical properties of temperature-sensitive PVA-PNIPAm hydrogel change rapidly from 28 to 34 °C, which can be used as a trigger in various ultrasonic applications. The compression tested Young’s Modulus of PVA-PNIPAm increases abruptly between 28 and 34 °C, whereas the Poisson’s ratio decreases from 31 to 32 °C. From 22 to 39 °C, the de-swelling ratio due to volumetric phase transition dropped from 100% to 30%. The novel temperature and frequency-dependent dynamic bulk modulus demonstrated that the dynamic elasticity of the PVA-PNIPAm is dependent not only on the temperature or the frequency but also on the co-dependence of the two factors. By changing the temperature and operating frequency, the 174% tunable range of the dynamic bulk modulus could offer great potential for ultrasonic applications. The temperature and frequency dynamic elasticity contour of PVA-PNIPAm in this study provides a materials and response co-relationship for the design and property selection of future acoustic applications.

## Appendix A

**Table A1:** Normalization factors for Figure 3 and Figure 5.

	Figure 3A	Figure 3B	Figure 3C	Figure 3D	Figure 5
PVA-PNIPAm	14.78 kPa	0.46	61.583 kPa	1048.9 kg m^−3^	1.650 GPa
6% Alginate	25.694 kPa	0.394	9.857 kPa	1257.3 kg m^−3^	2.024 GPa
10% Alginate	65.146 kPa	0.375	23.689 kPa	1409.2 kg m^−3^	2.877 GPa
14% Alginate	151.759 kPa	0.355	55.999 kPa	1410.8 kg m^−3^	3.005 GPa
